# Supplementary material for: A missense in HSF2BP causing primary ovarian insufficiency affects meiotic recombination by its novel interactor C19ORF57/BRME1
Source: eLife. 2020 Aug 26;9:e56996. doi: 10.7554/eLife.56996 (PMC7498267; doi:10.7554/eLife.56996)
Supplement: Supplementary file 1. — Supplementary file 1a. shows the whole exome sequencing and mapping metrics for the three genomic samples. Supplementary file 1b. shows the numbers of variants from the WES analysis and passing the various filters. Supplementary file 1c. shows the predictions of pathogenicity and conservations by 18 computational predictors. Supplementary files 1d and 1e. show the quantification of the colocalization between HSF2BP, BRME1, RPA1 and DMC1. Supplementary file 1f. shows the comparative alterations between all the mutants. Associated to Figures 4, 5, 6 and 9. Supplementary files 1g and 1h. show the putative BRME1 interactors identified by mass spectrometry. Supplementary files 1i and 1j. show respectively the crRNAs and the ssODN employed in the generation of the different mouse models. Supplementary file 1k. shows the primers and expected product sizes for genotyping mouse models. Supplementary file 1l. shows a summary of all the main and supplementary figures and their relationship. [file elife-56996-supp1.docx]

**Supplementary File 1**

**Supplementary file 1a. Whole Exome Sequencing and mapping data for the POI patients and the unaffected sister.**

|  | Read Pairs (millions) | Mean Depth | Coverage  > 25 X | Mismatch Read 1 | Mismatch Read 2 |
| --- | --- | --- | --- | --- | --- |
| POI III-2 | 56.2 | 79.05 X | 80.1 % | 0.13 % | 0.23 % |
| POI III-3 | 36.5 | 26.81 X | 89.86 % | 0.15 % | 0.29 % |
| NA III-10 | 31.1 | 38.39X | 92.1 % | 0.16 % | 0.25 % |

**Supplementary file 1b. Filtering of the variants identified by Whole Exome Sequencing.**

| Variants called in | POI III-2 | POI III-3 | NA III-10 |  |  |
| --- | --- | --- | --- | --- | --- |
| Total | **118545** | **105899** | **107840** |  |  |
| SNPs | 108106 | 96854 | 98621 |  |  |
| Indels | 10439 | 9045 | 9219 |  |  |
| Homozygous variants | 42695 | 39741 | 39640 |  |  |
| Heterozygous variants | 75850 | 66158 | 68200 |  |  |
| Variant filters | | | | | **# of variants** |
| Coverage > 9 reads in the 3 exomes | | | | | 130021 |
| Homozygous in patients, Homozygous Ref or Heterozygous in non-affected sister | | | | | 8897 |
| In coding regions | | | | | 4553 |
| With protein impact (i.e not synonymous) | | | | | 2295 |
| not Homozygous in unrelated fertile controls | | | | | 66 |
| MAF < 1% in GnomAD | | | | | 5 |
| with coherent functional information | | | | | 1 |

**Supplementary file 1c: Pathogenicity predictions for the S167L variant in *HSF2BP.***

| Software | Score for the variant | Pathogenicity threshold | Pathogenicity  prediction |
| --- | --- | --- | --- |
| SIFT | 0.114 | < 0.05 | Tolerated |
| SIFT4G | 0.004 | < 0.05 | Damaging |
| PolyPhen 2 | 0.135 | > 0.8 | Benign (indicated as  probably_damaging in GnomAD |
| M-CAP | 0.0232 | > 0.025 | Tolerated (borderline) |
| FATHMM-MKL | 0.8975 | > 0.5 | Damaging |
| LRT | 0.000293 | Score is a p-value | Deleterious |
| MutationTaster | 0.994943 | 0.5 | Disease-causing |
| MutationAssessor | 1.87 | > 0.65 | Low |
| FATHMM | -0.06 | < -1.5 | Tolerated |
| FATHMM-MKL coding | 0.89755 | 0.5 (default) 0.80 (stringent) | Deleterious |
| PROVEAN | -2.88 | -2.28 | Damaging |
| MetaSVM | -0.8926 | 0 | Tolerated |
| MetaLR | 0.1613 | 0.5 | Tolerated |
| REVEL | 0.068 | 0.5 (default) 0.75 (stringent) | Benign |
| DANN | 0.996469 | 0.96 | Damaging |
| CADD | 5.470242 | 1.75 | Damaging |
| GERP++ RS | 4.36 | > 4.4 | Conserved |
| phyloP100way_vertebrate | 5.335 | > 1.6 | Highly conserved |

**Supplementary file 1d. Quantification of HSF2BP colocalization with RPA1, DMC1 and BRME1.** The quantification was carried out from three cells corresponding to each stage. Non-specific colocalization (random colocalization) was quantified in the same cells but after rotating one of the images 90 degrees.

| STAGE | HSF2BP foci stained positive for BRME1 | | BRME1 foci stained positive for HSF2BP | |
| --- | --- | --- | --- | --- |
|  | **Specific**  **(w/o rotation)** | **Non-specific**  **(with rotation)** | **Specific**  **(w/o rotation)** | **Specific**  **(w/o rotation)** |
| **Early zygotene** | 91,5% ± 2,0 | 4,1% ± 1,3 | 68,7% ± 1,0 | 3,1% ± 1,0 |
| **Late zygotene** | 94,1% ± 1,2 | 3,1% ± 0,7 | 88,0% ± 3,0 | 2,9% ± 0,7 |
| **Early pachytene** | 92,6% ± 2,6 | 2,8% ± 1,7 | 89,6% ± 2,8 | 2,7% ± 1,7 |

| STAGE | HSF2BP foci stained positive for DMC1 | | DMC1 foci stained positive for HSF2BP | |
| --- | --- | --- | --- | --- |
|  | **Specific**  **(w/o rotation)** | **Non-specific**  **(with rotation)** | **Specific**  **(w/o rotation)** | **Non-specific**  **(with rotation)** |
| **Early zygotene** | 74,0% ± 11,1 | 1,0% ± 0,5 | 87,3% ± 8,1 | 1,2% ± 0,7 |
| **Late zygotene** | 55,3% ± 13,4 | 1,9% ± 1,1 | 89,1% ± 6,1 | 3,3% ± 2,5 |

| STAGE | HSF2BP foci stained positive for RPA1 | | RPA1 foci stained positive for HSF2BP | |
| --- | --- | --- | --- | --- |
|  | **Specific**  **(w/o rotation)** | **Non-specific**  **(with rotation)** | **Specific**  **(w/o rotation)** | **Specific**  **(w/o rotation)** |
| **Early zygotene** | 76,3% ± 2,0 | 5,5% ± 4,4 | 69,2% ± 0,8 | 5,0% ± 4,1 |
| **Late zygotene** | 81,8% ± 3,1 | 3,0% ± 0,9 | 82,4% ± 3,4 | 3,0% ± 1,0 |
| **Early pachytene** | 79,7% ± 3,5 | 2,8% ± 1,7 | 88,6% ± 3,5 | 3,1% ± 2,0 |

**Supplementary file 1e. Quantification of BRME1 colocalization with RPA2 and DMC1.** The quantification was carried out from three cells corresponding to each stage. Non-specific colocalization (random colocalization) was quantified in the same cells but after rotating one of the images 90 degrees.

| STAGE | BRME1 foci stained positive for RPA2 | | RPA2 foci stained positive for BRME1 | |
| --- | --- | --- | --- | --- |
|  | **Specific**  **(w/o rotation)** | **Non-specific**  **(with rotation)** | **Specific**  **(w/o rotation)** | **Non-specific**  **(with rotation)** |
| **Early zygotene** | 90,5% ± 2,3 | 2,2% ± 0,9 | 84,6 ± 3,9 | 2,1% ± 0,8 |
| **Late zygotene** | 90,2% ± 1,5 | 2,0% ± 0,4 | 95,2% ± 0,6 | 2,1% ± 0,4 |
| **Early pachytene** | 87,7% ± 5,0 | 1,8% ± 0,2 | 92,4% ± 4,8 | 1,9% ± 0,3 |

| STAGE | BRME1 foci stained positive for DMC1 | | DMC1 foci stained positive for BRME1 | |
| --- | --- | --- | --- | --- |
|  | **Specific**  **(w/o rotation)** | **Non-specific**  **(with rotation)** | **Specific**  **(w/o rotation)** | **Non-specific**  **(with rotation)** |
| **Early zygotene** | 58,3% ± 4,6 | 5,1% ± 2,4 | 55,5% ± 5,0 | 4,9% ± 2,5 |
| **Late zygotene** | 30,8% ± 2,6 | 2,7% ± 1,0 | 78,8% ± 13,7 | 7,0% ± 1,7 |

| **Supplementary file 1f. Summary table comparing the different alterations in the meiotic recombination pathway on each genotype (both sexes) for the different meiotic players analyzed (γH2AX, RPA1, SPATA22, DMC1, RAD51 and MLH1).** |  |  | Lepto/Early zygo | Zygo/Zygo-like | Pachy |
| --- | --- | --- | --- | --- | --- |
| γH2AX | *Hsf2bp*^S167L/S167L^ | males |  |  | **>>** |
|  |  | females |  |  | **>** |
|  | *Hsf2bp^-/-^* | males |  | ∞ | N/A |
|  |  | females |  |  | **>>>** |
|  | *Brme1^-/-^* | males |  | ∞ | N/A |
|  |  | females |  |  | **>>** |
| RPA1 | *Hsf2bp*^S167L/S167L^ | males | **=** | **=** | **>>** |
|  |  | females |  | **=** | **=** |
|  | *Hsf2bp^-/-^* | males | **=** | **>** | N/A |
|  |  | females |  | **=** | **=** |
|  | *Brme1^-/-^* | males | **=** | **>** | N/A |
|  |  | females |  | **=** | **=** |
| SPATA22 | *Hsf2bp*^S167L/S167L^ | males |  | **>** | **>** |
|  |  | females |  | **>? ns** | **>? ns** |
|  | *Hsf2bp^-/-^* | males |  | **>>** | N/A |
|  |  | females |  | **>>** | **>>** |
|  | *Brme1^-/-^* | males |  | >>> | N/A |
|  |  | females |  | **>>** | **>>>** |
| DMC1 | *Hsf2bp*^S167L/S167L^ | males | **<** | **-?ns** | **<** |
|  |  | females |  | **<<** | **<<** |
|  | *Hsf2bp^-/-^* | males | **<<<** | **<<<** | N/A |
|  |  | females |  | **<<<** | **<<<** |
|  | *Brme1^-/-^* | males | **<<<** | **<<<** | N/A |
|  |  | females |  | **<<<** | **<<<** |
|  | | | | | |
|  | | | | | |
|  |  |  | **Lepto/Early zygo** | **Zygo/Zygo-like** | **Pachy** |
| RAD51 | *Hsf2bp*^S167L/S167L^ | males | **<<** | **<<** | **<** |
|  |  | females |  | **<<** | **<<<** |
|  | *Hsf2bp^-/-^* | males | **<<<** | <<< | N/A |
|  |  | females |  | **<<<** | **<<<** |
|  | *Brme1^-/-^* | males | **<<** | **<<<** | N/A |
|  |  | females |  | **<<** | **<<<** |
|  | | | **Total foci** | **% cells w/o foci in XY** | **% cells w/o foci in autosomes** |
| MLH1 | Wild type | males | 25 | 27% | 3% |
|  |  | females | 24 | N/A | 21% |
|  | *Hsf2bp*^S167L/S167L^ | males | 21 | 55% | 20% |
|  |  | females | 22 | N/A | 48% |
|  | *Hsf2bp^-/-^* | males | 0 | N/A | N/A |
|  |  | females | 19 | N/A | 75% |
|  | *Brme1^-/-^* | males | 0 | N/A | N/A |
|  |  | females | 19 | N/A | 79% |

**The wild type mean has been considered as the reference value:**

**>** : Higher than wild type

**<** : Lower than wild type

= : Similar to wild type

∞: The signal is similar in zygotene-like arrested cells and in wild type zygotene cells, but in the arrested cells it remains permanently.

ns: non-significant differences (Two-tailed Welch´s t-test analysis).

N/A: Not Applicable due to a previous meiotic arrest in *Hsf2bp^-/-^* and *Brme1^-/-^* males or due to the absence of XY bivalent in females (MLH1 data).

Empty cells: not tested.

| Gene names | Protein IDs | R1-Num of unique peptides | R2- Num of unique peptides | Control-Num of unique peptides | R1 iBAQ | R2 iBAQ | Control iBAQ | Group |
| --- | --- | --- | --- | --- | --- | --- | --- | --- |
| Hsf2bp | Q9D4G2 | 20 | 16 | 0 | 168840000 | 37607000 | 0 | R1 and R2 only |
| C19orf57  (Brme1) | Q6DIA7 | 16 | 13 | 1 | 77473000 | 22181000 | 59511 | R1 and R2 only |
| Brca2 | P97929 | 78 | 77 | 0 | 5296400 | 3125000 | 0 | R1 and R2 only |
| Palb2 | Q3U0P1 | 7 | 11 | 0 | 1102300 | 896460 | 0 | R1 and R2 only |
| Rad51 | Q08297 | 2 | 4 | 0 | 509370 | 367390 | 0 | R1 and R2 only |
| Rpa2 | Q62193 | 3 | 0 | 0 | 235990 | 0 | 0 | R1 only |

**Supplementary file 1g. Meiotic recombination players identified as putative BRME1 interactors by immunoprecipitation coupled to mass spectrometry of whole testis extracts with two antibodies (R1 and R2) against BRME1.** See extended data on supplementary file 1h.

**Supplementary file 1h. Extended data of BRME1 putative interactors identified by immunoprecipitation coupled to mass spectrometry of whole testis extracts with two different antibodies (R1 and R2) against BRME1 (see Methods).**

| Gene names | Protein IDs | R1-Num of unique peptides | R2-Num of unique peptides | Control-Num of unique peptides | R1 iBAQ | R2 iBAQ | Control iBAQ | Group |
| --- | --- | --- | --- | --- | --- | --- | --- | --- |
| Hsf2bp | Q9D4G2 | 20 | 16 | 0 | 168840000 | 37607000 | 0 | R1 and R2 only |
| Edc4 | Q3UJB9 | 3 | 46 | 1 | 193300 | 24305000 | 59266 | R1 and R2 only |
| C19orf57  (Brme1) | Q6DIA7 | 16 | 13 | 1 | 77473000 | 22181000 | 59511 | R1 and R2 only |
| Dhrs11 | Q3U0B3 | 3 | 11 | 0 | 424760 | 10761000 | 0 | R1 and R2 only |
| Rpl27 | P61358 | 4 | 3 | 1 | 14163000 | 6680600 | 189540 | R1 and R2 only |
| Ccar2 | Q8VDP4 | 24 | 22 | 0 | 10790000 | 5672600 | 0 | R1 and R2 only |
| Sec13 | Q9D1M0 | 10 | 9 | 0 | 4987100 | 5509600 | 0 | R1 and R2 only |
| Dynll2 | Q9D0M5 | 3 | 3 | 1 | 18915000 | 4495000 | 449620 | R1 and R2 only |
| Rpl18 | P35980 | 3 | 4 | 0 | 6704400 | 4425100 | 0 | R1 and R2 only |
| Usp4 | P35123;Q99K46 | 37 | 24 | 0 | 40187000 | 3667700 | 0 | R1 and R2 only |
| Brca2 | P97929 | 78 | 77 | 0 | 5296400 | 3125000 | 0 | R1 and R2 only |
| Hspd1 | P63038 | 8 | 11 | 1 | 1885200 | 2681000 | 43682 | R1 and R2 only |
| Rpl10a | P53026 | 6 | 3 | 0 | 27684000 | 2361300 | 0 | R1 and R2 only |
| Chtf8 | P0CG14 | 17 | 16 | 0 | 4464800 | 1811600 | 0 | R1 and R2 only |
| Rpl13a | P19253 | 3 | 3 | 0 | 7370600 | 1474600 | 0 | R1 and R2 only |
| Rpl7 | P14148 | 10 | 4 | 1 | 8627200 | 1416200 | 188040 | R1 and R2 only |
| Dnajb11 | Q99KV1 | 3 | 6 | 1 | 1009800 | 1414900 | 137790 | R1 and R2 only |
| Rpl6 | P47911 | 7 | 5 | 1 | 4884800 | 1182700 | 41048 | R1 and R2 only |
| Rpl30 | P62889 | 4 | 4 | 0 | 3876500 | 1174300 | 0 | R1 and R2 only |
| Arhgef2 | Q60875 | 0 | 12 | 0 | 0 | 942440 | 0 | R2 only |
| Palb2 | Q3U0P1 | 7 | 11 | 0 | 1102300 | 896460 | 0 | R1 and R2 only |
| Wnk1 | P83741;Q80UE6 | 16 | 9 | 0 | 2979600 | 835530 | 0 | R1 and R2 only |
| Rpl27a | P14115 | 2 | 1 | 0 | 4934400 | 768520 | 0 | R1 only |
| Rpl8 | P62918 | 3 | 3 | 1 | 4026000 | 754840 | 53677 | R1 and R2 only |
| Exosc5 | Q9CRA8 | 2 | 3 | 0 | 492230 | 714900 | 0 | R1 and R2 only |
| Tex33 | Q9D9J2 | 4 | 4 | 1 | 2079400 | 686300 | 249520 | R1 and R2 only |
| Bag5 | Q8CI32 | 3 | 10 | 0 | 98819 | 677230 | 0 | R1 and R2 only |
| Rpap3 | Q9D706 | 0 | 8 | 0 | 0 | 624360 | 0 | R2 only |
| Ybx1 | P62960 | 4 | 3 | 1 | 1559000 | 610700 | 36539 | R1 and R2 only |
| Ilf2 | Q9CXY6 | 7 | 4 | 1 | 1504400 | 592600 | 11488 | R1 and R2 only |
| Tdrd1 | Q99MV1 | 2 | 10 | 0 | 86505 | 561490 | 0 | R1 and R2 only |
| Ctnnb1 | Q02248 | 0 | 6 | 0 | 0 | 533930 | 0 | R2 only |
| Arhgdia | Q99PT1 | 2 | 3 | 1 | 673000 | 506600 | 145900 | R1 and R2 only |
| Rpl32 | P62911 | 3 | 3 | 0 | 1224000 | 479360 | 0 | R1 and R2 only |
| Ybx2 | Q9Z2C8 | 7 | 4 | 1 | 4297400 | 477700 | 156530 | R1 and R2 only |
| Dnaja4 | Q9JMC3 | 4 | 7 | 1 | 729390 | 461060 | 54926 | R1 and R2 only |
| Rpl24 | Q8BP67 | 3 | 2 | 0 | 2133500 | 458720 | 0 | R1 and R2 only |
| Hspa1l | P16627 | 2 | 2 | 0 | 684830 | 438960 | 0 | R1 and R2 only |
| Cenpj | Q569L8 | 11 | 11 | 0 | 544000 | 423970 | 0 | R1 and R2 only |
| Rpl4 | Q9D8E6 | 6 | 3 | 0 | 1783600 | 410860 | 0 | R1 and R2 only |
| Ewsr1 | Q61545 | 4 | 3 | 1 | 3332800 | 408390 | 74676 | R1 and R2 only |
| Rpl28 | P41105 | 2 | 2 | 0 | 1146400 | 385370 | 0 | R1 and R2 only |
| Rbm14 | Q8C2Q3 | 6 | 5 | 0 | 1316300 | 378870 | 0 | R1 and R2 only |
| Eif4e | P63073 | 3 | 2 | 1 | 1875700 | 375610 | 71180 | R1 and R2 only |
| Aifm1 | Q9Z0X1 | 1 | 6 | 0 | 99312 | 374800 | 0 | R2 only |
| Rps8 | P62242 | 5 | 2 | 0 | 2807000 | 369660 | 0 | R1 and R2 only |
| Rad51 | Q08297 | 2 | 4 | 0 | 509370 | 367390 | 0 | R1 and R2 only |
| Xlr3c;Xlr3b;Xlr3a | Q61806;Q6P205;Q60595 | 1 | 3 | 0 | 58041 | 367380 | 0 | R2 only |
| Ranbp1 | P34022 | 0 | 2 | 0 | 0 | 365060 | 0 | R2 only |
| Vapa | Q9WV55 | 5 | 2 | 0 | 2393500 | 359790 | 0 | R1 and R2 only |
| Amy1;Amy2 | P00687;P00688 | 2 | 2 | 0 | 4730900 | 355380 | 0 | R1 and R2 only |
| Echs1 | Q8BH95 | 3 | 3 | 1 | 1090100 | 347120 | 180080 | R1 and R2 only |
| Dnaja2 | Q9QYJ0 | 5 | 4 | 0 | 508220 | 329450 | 0 | R1 and R2 only |
| Rpl19 | P84099 | 2 | 2 | 0 | 2200900 | 329190 | 0 | R1 and R2 only |
| Fahd2 | Q3TC72 | 3 | 2 | 1 | 659830 | 326080 | 90862 | R1 and R2 only |
| Exosc4 | Q921I9 | 3 | 2 | 0 | 159550 | 320140 | 0 | R1 and R2 only |
| Glt8d1 | Q6NSU3 | 8 | 2 | 1 | 3636300 | 315810 | 8110,6 | R1 and R2 only |
| Eif2b4 | Q61749 | 0 | 3 | 0 | 0 | 308760 | 0 | R2 only |
| Exosc2 | Q8VBV3 | 3 | 4 | 0 | 225990 | 294010 | 0 | R1 and R2 only |
| Bag2 | Q91YN9 | 1 | 3 | 0 | 301170 | 291950 | 0 | R2 only |
| Hnrnpm | Q9D0E1 | 4 | 6 | 0 | 476840 | 286690 | 0 | R1 and R2 only |
| Anxa1 | P10107 | 4 | 4 | 1 | 599940 | 274100 | 16392 | R1 and R2 only |
| Kif21a | Q9QXL2;Q9QXL1;B7ZNG0 | 0 | 8 | 0 | 0 | 263910 | 0 | R2 only |
| Sorbs3 | Q9R1Z8 | 0 | 3 | 0 | 0 | 260670 | 0 | R2 only |
| Rpgr | Q9R0X5 | 0 | 4 | 0 | 0 | 258990 | 0 | R2 only |
| Psmc4 | P54775 | 5 | 4 | 1 | 450370 | 255350 | 17166 | R1 and R2 only |
| Caprin1 | Q60865 | 6 | 2 | 0 | 1890700 | 254830 | 0 | R1 and R2 only |
| Pcbp2 | Q61990 | 4 | 2 | 1 | 1067500 | 252810 | 148480 | R1 and R2 only |
| Pgrmc1 | O55022 | 1 | 2 | 0 | 181560 | 241760 | 0 | R2 only |
| Eif6 | O55135 | 5 | 2 | 0 | 2835600 | 237780 | 0 | R1 and R2 only |
| Mamld1 | P0C6A2 | 1 | 4 | 0 | 75258 | 236850 | 0 | R2 only |
| Rpl21 | O09167 | 1 | 2 | 0 | 648660 | 225840 | 0 | R2 only |
| Rps24 | P62849 | 1 | 2 | 0 | 591820 | 223810 | 0 | R2 only |
| Exosc6 | Q8BTW3 | 1 | 2 | 0 | 186290 | 213080 | 0 | R2 only |
| Wdr34 | Q5U4F6 | 1 | 3 | 0 | 186700 | 211000 | 0 | R2 only |
| Rpgrip1 | Q9EPQ2 | 0 | 4 | 0 | 0 | 196320 | 0 | R2 only |
| Hagh | Q99KB8 | 6 | 4 | 0 | 1000700 | 193140 | 0 | R1 and R2 only |
| Gapvd1 | Q6PAR5 | 0 | 6 | 0 | 0 | 182050 | 0 | R2 only |
| Slc2a3 | P32037 | 5 | 2 | 1 | 4020900 | 180730 | 282890 | R1 and R2 only |
| Pfkl | P12382 | 1 | 4 | 0 | 57749 | 176310 | 0 | R2 only |
| Rbm17 | Q8JZX4 | 1 | 4 | 0 | 26197 | 173970 | 0 | R2 only |
| Eif2b5 | Q8CHW4 | 0 | 2 | 0 | 0 | 169930 | 0 | R2 only |
| Dnajb6 | O54946;Q9QYI8 | 1 | 2 | 0 | 195800 | 163940 | 0 | R2 only |
| Tprn | A2AI08 | 0 | 2 | 0 | 0 | 162190 | 0 | R2 only |
| Ropn1l | Q9EQ00 | 2 | 1 | 0 | 697100 | 156610 | 0 | R1 only |
| Npm1 | Q61937 | 2 | 2 | 1 | 296120 | 154640 | 38063 | R1 and R2 only |
| Ddx5;Ddx17 | Q61656;Q501J6 | 2 | 2 | 1 | 704920 | 150620 | 70359 | R1 and R2 only |
| Lyar | Q08288 | 2 | 2 | 1 | 154100 | 148270 | 29145 | R1 and R2 only |
| Tbl1xr1 | Q8BHJ5 | 0 | 3 | 0 | 0 | 140050 | 0 | R2 only |
| Pfkp | Q9WUA3 | 5 | 3 | 0 | 382140 | 139640 | 0 | R1 and R2 only |
| Clcc1 | Q99LI2 | 0 | 4 | 0 | 0 | 132410 | 0 | R2 only |
| Ubap2l | Q80X50 | 1 | 3 | 0 | 66946 | 130510 | 0 | R2 only |
| Vapb | Q9QY76 | 2 | 2 | 0 | 461320 | 125430 | 0 | R1 and R2 only |
| Dlst | Q9D2G2 | 2 | 2 | 1 | 235820 | 121910 | 112620 | R1 and R2 only |
| Ndel1 | Q9ERR1;Q9CZA6 | 0 | 3 | 0 | 0 | 120480 | 0 | R2 only |
| Htra2 | Q9JIY5 | 0 | 2 | 0 | 0 | 119120 | 0 | R2 only |
| Polr2e | Q80UW8 | 0 | 2 | 0 | 0 | 115740 | 0 | R2 only |
| Rbm7 | Q9CQT2 | 2 | 2 | 0 | 176710 | 115380 | 0 | R1 and R2 only |
| Psma8;Psma7 | Q9CWH6;Q9Z2U0 | 5 | 2 | 1 | 1189900 | 111700 | 113360 | R1 and R2 only |
| Stk39 | Q9Z1W9 | 4 | 3 | 0 | 564060 | 110480 | 0 | R1 and R2 only |
| Pld3 | O35405 | 2 | 1 | 0 | 546090 | 107090 | 0 | R1 only |
| Eif2b2 | Q99LD9 | 0 | 3 | 0 | 0 | 106890 | 0 | R2 only |
| Tdrd6 | P61407 | 11 | 9 | 0 | 461070 | 106510 | 0 | R1 and R2 only |
| Shcbp1l | Q3TTP0 | 3 | 2 | 1 | 179970 | 106190 | 14360 | R1 and R2 only |
| Tarbp2 | P97473 | 0 | 2 | 0 | 0 | 105740 | 0 | R2 only |
| Spata20 | Q80YT5 | 4 | 3 | 0 | 410910 | 104640 | 0 | R1 and R2 only |
| Trafd1 | Q3UDK1 | 1 | 2 | 0 | 0 | 104460 | 0 | R2 only |
| Serpina1b | P22599 | 2 | 0 | 0 | 2856200 | 102550 | 678480 | R1 only |
| Hprt1 | P00493 | 3 | 2 | 1 | 354350 | 102310 | 79077 | R1 and R2 only |
| Hnrnpk | P61979 | 5 | 1 | 0 | 512010 | 100340 | 0 | R1 only |
| Strap | Q9Z1Z2 | 9 | 3 | 0 | 874350 | 98155 | 0 | R1 and R2 only |
| C1sa;C1sb | Q8CG14;Q8CFG8 | 4 | 2 | 0 | 312700 | 95781 | 0 | R1 and R2 only |
| Akap12 | Q9WTQ5 | 5 | 4 | 0 | 241290 | 95498 | 0 | R1 and R2 only |
| Kif27 | Q7M6Z4 | 0 | 6 | 0 | 0 | 95153 | 0 | R2 only |
| Stk31 | Q99MW1 | 4 | 4 | 1 | 133550 | 93796 | 5679,5 | R1 and R2 only |
| Sec23a;Sec23b | Q01405;Q9D662 | 2 | 4 | 0 | 108710 | 92427 | 0 | R1 and R2 only |
| Lrrc34 | Q9DAM1 | 4 | 4 | 0 | 68517 | 89375 | 0 | R1 and R2 only |
| Hip1 | Q8VD75 | 0 | 3 | 0 | 0 | 87633 | 0 | R2 only |
| Exosc7 | Q9D0M0 | 0 | 2 | 0 | 0 | 87082 | 0 | R2 only |
| Stk36 | Q69ZM6 | 0 | 3 | 0 | 0 | 86558 | 0 | R2 only |
| Dkkl1 | Q9QZL9 | 1 | 2 | 0 | 84507 | 86106 | 0 | R2 only |
| Sdhb | Q9CQA3 | 5 | 2 | 0 | 821740 | 85886 | 0 | R1 and R2 only |
| Mael | Q8BVN9 | 2 | 3 | 1 | 333530 | 80932 | 40646 | R1 and R2 only |
| Hspa4 | Q61316 | 3 | 3 | 0 | 260230 | 80515 | 0 | R1 and R2 only |
| Fam184b | Q0KK56 | 0 | 4 | 0 | 0 | 79391 | 0 | R2 only |
| Atxn2l | Q7TQH0 | 7 | 2 | 0 | 960960 | 78810 | 0 | R1 and R2 only |
| Atp5b | P56480 | 4 | 3 | 1 | 248730 | 75502 | 17119 | R1 and R2 only |
| Als2 | Q920R0 | 0 | 4 | 0 | 0 | 73670 | 0 | R2 only |
| Fn1 | P11276 | 0 | 6 | 0 | 0 | 70072 | 0 | R2 only |
| Cope | O89079 | 1 | 2 | 0 | 70934 | 68740 | 0 | R2 only |
| Nup133 | Q8R0G9 | 0 | 3 | 0 | 0 | 68014 | 0 | R2 only |
| Ruvbl2 | Q9WTM5 | 0 | 2 | 0 | 0 | 67203 | 0 | R2 only |
| Tekt4 | Q149S1 | 1 | 2 | 0 | 54604 | 65884 | 0 | R2 only |
| Rtcb | Q99LF4 | 3 | 2 | 1 | 415760 | 65057 | 66810 | R1 and R2 only |
| Lancl2 | Q9JJK2 | 4 | 2 | 0 | 229810 | 63780 | 0 | R1 and R2 only |
| Pygm;Pygb | Q9WUB3;Q8CI94;Q9ET01 | 2 | 2 | 1 | 53539 | 61107 | 13916 | R1 and R2 only |
| Eif3k | Q9DBZ5 | 2 | 1 | 0 | 303390 | 60567 | 0 | R1 only |
| Hspbp1 | Q99P31 | 0 | 2 | 0 | 0 | 59812 | 0 | R2 only |
| Dpysl3;Crmp1 | Q62188;P97427 | 2 | 1 | 0 | 0 | 58906 | 0 | R1 only |
| Snrpb;Snrpn | P27048;P63163 | 4 | 1 | 0 | 665630 | 58830 | 0 | R1 only |
| Atp2a2 | O55143;Q64518;Q8R429 | 4 | 3 | 0 | 338550 | 58794 | 0 | R1 and R2 only |
| Acly | Q91V92 | 1 | 4 | 0 | 28857 | 56271 | 0 | R2 only |
| Sox30 | Q8CGW4 | 0 | 2 | 0 | 0 | 55897 | 0 | R2 only |
| Kif5b | Q61768;P28738;P33175 | 11 | 3 | 1 | 595910 | 54255 | 20536 | R1 and R2 only |
| Hnrnpa3 | Q8BG05 | 3 | 2 | 0 | 442780 | 53115 | 0 | R1 and R2 only |
| Clgn | P52194 | 3 | 2 | 1 | 344620 | 49523 | 62274 | R1 and R2 only |
| Skt | A2AQ25 | 0 | 5 | 0 | 0 | 48382 | 0 | R2 only |
| Rqcd1 | Q9JKY0 | 2 | 1 | 0 | 91131 | 47676 | 0 | R1 only |
| Gmps | Q3THK7 | 2 | 2 | 0 | 131990 | 46712 | 0 | R1 and R2 only |
| D1Pas1;Ddx3x;Ddx3y | P16381;Q62167;Q62095 | 2 | 2 | 0 | 149500 | 46676 | 0 | R1 and R2 only |
| Odf1 | Q61999 | 2 | 1 | 0 | 332760 | 44049 | 0 | R1 only |
| Ruvbl1 | P60122 | 0 | 2 | 0 | 0 | 43574 | 0 | R2 only |
| Kifap3 | P70188 | 2 | 1 | 0 | 231490 | 41384 | 0 | R1 only |
| Actl7a | Q9QY84 | 0 | 2 | 0 | 0 | 40644 | 0 | R2 only |
| Ubr5 | Q80TP3 | 0 | 6 | 0 | 0 | 38908 | 0 | R2 only |
| Rock1 | P70335;P70336 | 0 | 3 | 0 | 0 | 38476 | 0 | R2 only |
| Rps2 | P25444 | 5 | 1 | 0 | 1473300 | 38357 | 0 | R1 only |
| RtcA | Q9D7H3 | 2 | 1 | 0 | 114410 | 37842 | 0 | R1 only |
| Kiaa1324 | A2AFS3 | 10 | 1 | 0 | 5900000 | 37639 | 0 | R1 only |
| Apoa1bp | Q8K4Z3 | 2 | 1 | 0 | 246830 | 36405 | 0 | R1 only |
| Txnrd3 | Q99MD6 | 7 | 1 | 0 | 1342700 | 36342 | 0 | R1 only |
| Tbc1d1 | Q60949 | 0 | 3 | 0 | 0 | 36202 | 0 | R2 only |
| Timm50 | Q9D880 | 2 | 1 | 0 | 173140 | 35819 | 0 | R1 only |
| Blvra | Q9CY64 | 3 | 2 | 1 | 287230 | 35317 | 26522 | R1 and R2 only |
| Cpsf1 | Q9EPU4 | 7 | 3 | 0 | 407640 | 34499 | 0 | R1 and R2 only |
| Flna | Q8BTM8 | 1 | 5 | 0 | 9298,2 | 34102 | 0 | R2 only |
| Ccdc13 | D3YV10 | 2 | 2 | 0 | 65841 | 33416 | 0 | R1 and R2 only |
| Fyttd1 | Q91Z49 | 3 | 1 | 0 | 412970 | 33406 | 0 | R1 only |
| Poldip3 | Q8BG81 | 2 | 2 | 0 | 45321 | 31020 | 0 | R1 and R2 only |
| Piwil1 | Q9JMB7 | 10 | 3 | 0 | 767380 | 30549 | 0 | R1 and R2 only |
| Tpp2 | Q64514 | 6 | 2 | 0 | 219590 | 28832 | 0 | R1 and R2 only |
| Kif3a | P28741;P33174 | 3 | 1 | 0 | 160620 | 28635 | 0 | R1 only |
| Uso1 | Q9Z1Z0 | 0 | 2 | 0 | 0 | 28261 | 0 | R2 only |
| Vim | P20152;P31001 | 2 | 2 | 0 | 53445 | 26731 | 0 | R1 and R2 only |
| Tln1 | P26039;Q71LX4 | 5 | 5 | 0 | 68758 | 26539 | 0 | R1 and R2 only |
| Pc | Q05920 | 2 | 1 | 0 | 98436 | 26509 | 0 | R1 only |
| Hsp90R1 | P11499 | 5 | 1 | 0 | 1037800 | 26372 | 0 | R1 only |
| Map4 | P27546 | 1 | 2 | 0 | 67052 | 25354 | 0 | R2 only |
| Upf1 | Q9EPU0 | 18 | 2 | 0 | 2328000 | 25293 | 0 | R1 and R2 only |
| Golga1 | Q9CW79 | 0 | 2 | 0 | 0 | 22905 | 0 | R2 only |
| Myh8;Myh4;Myh1 | P13542;Q5SX39;Q5SX40 | 2 | 2 | 0 | 44360 | 22133 | 0 | R1 and R2 only |
| Hnrnpu | Q8VEK3 | 3 | 1 | 0 | 396380 | 22088 | 0 | R1 only |
| Ahsa1 | Q8BK64 | 2 | 1 | 0 | 171790 | 21464 | 0 | R1 only |
| Uhrf1bp1l | A2RSJ4 | 7 | 1 | 0 | 442990 | 21354 | 0 | R1 only |
| Pgam5 | Q8BX10 | 3 | 1 | 0 | 504060 | 21146 | 0 | R1 only |
| Man2c1 | Q91W89 | 2 | 2 | 1 | 63138 | 20969 | 12134 | R1 and R2 only |
| Tjp1 | P39447 | 10 | 4 | 0 | 426880 | 20721 | 0 | R1 and R2 only |
| Eif4a3 | Q91VC3 | 2 | 1 | 0 | 52120 | 20420 | 0 | R1 only |
| Eci1 | P42125 | 2 | 1 | 0 | 107850 | 19520 | 0 | R1 only |
| Stat4 | P42228 | 1 | 2 | 0 | 18273 | 18517 | 0 | R2 only |
| Il4i1 | O09046 | 2 | 1 | 0 | 144390 | 16047 | 0 | R1 only |
| Prps1;Prps2 | Q9D7G0;Q9CS42 | 6 | 1 | 0 | 523570 | 14765 | 0 | R1 only |
| Xpnpep1 | Q6P1B1 | 2 | 1 | 0 | 63335 | 14765 | 0 | R1 only |
| Huwe1 | Q7TMY8 | 2 | 3 | 0 | 19703 | 13376 | 0 | R1 and R2 only |
| Ndufa10 | Q99LC3 | 2 | 1 | 0 | 41505 | 12590 | 0 | R1 only |
| Eprs | Q8CGC7 | 1 | 2 | 0 | 30547 | 12325 | 0 | R2 only |
| Kif3b;Kif3c | Q61771;O35066 | 6 | 2 | 0 | 329860 | 12146 | 0 | R1 and R2 only |
| Nup160 | Q9Z0W3 | 0 | 2 | 0 | 0 | 10456 | 0 | R2 only |
| Drg1 | P32233 | 3 | 1 | 0 | 128230 | 8231,4 | 0 | R1 only |
| Usp15 | Q8R5H1 | 3 | 1 | 0 | 101310 | 7650 | 0 | R1 only |
| Sf3a1 | Q8K4Z5 | 2 | 1 | 0 | 86356 | 5839,5 | 0 | R1 only |
| Vcl | Q64727 | 2 | 1 | 0 | 46706 | 5739,2 | 0 | R1 only |
| Cnot1 | Q6ZQ08 | 0 | 3 | 0 | 0 | 5397 | 0 | R2 only |
| Odf2 | A3KGV1 | 2 | 1 | 0 | 21144 | 2948,3 | 0 | R1 only |
| Dync1h1 | Q9JHU4 | 2 | 2 | 1 | 12517 | 843,22 | 5755,4 | R1 and R2 only |
| Tcea2 | Q9QVN7;P10711 | 15 | 0 | 0 | 77583000 | 0 | 0 | R1 only |
| Slc4a1 | P04919 | 17 | 0 | 0 | 35820000 | 0 | 0 | R1 only |
| Kif17 | Q99PW8;O35231 | 33 | 1 | 0 | 21062000 | 0 | 0 | R1 only |
| Cap2 | Q9CYT6 | 14 | 0 | 0 | 16491000 | 0 | 0 | R1 only |
| Kank3 | Q9Z1P7 | 15 | 0 | 0 | 8436100 | 0 | 0 | R1 only |
| Rpl9 | P51410 | 5 | 0 | 0 | 7949900 | 0 | 0 | R1 only |
| Dynll1 | P63168 | 3 | 0 | 0 | 6071100 | 0 | 0 | R1 only |
| Kctd9 | Q80UN1 | 9 | 0 | 0 | 3984300 | 0 | 0 | R1 only |
| Gtsf1 | Q9DAN6 | 3 | 0 | 0 | 2817800 | 0 | 0 | R1 only |
| Bcap29 | Q61334 | 3 | 0 | 0 | 2109700 | 0 | 0 | R1 only |
| Dnali1 | Q8BVN8 | 5 | 0 | 0 | 1832800 | 0 | 0 | R1 only |
| Ctnnd1 | P30999 | 12 | 0 | 0 | 1456600 | 0 | 0 | R1 only |
| Tmem239 | Q9DA47 | 2 | 0 | 0 | 1390700 | 0 | 0 | R1 only |
| Sh3d21 | Q7TSG5 | 6 | 0 | 0 | 1203800 | 0 | 0 | R1 only |
| Gnptg | Q6S5C2 | 4 | 0 | 0 | 1177600 | 0 | 0 | R1 only |
| Golga3 | P55937 | 20 | 0 | 0 | 1123200 | 0 | 0 | R1 only |
| Golga5 | Q9QYE6 | 10 | 0 | 0 | 988130 | 0 | 0 | R1 only |
| Pspc1 | Q8R326 | 2 | 0 | 0 | 960890 | 0 | 0 | R1 only |
| Rps13 | P62301 | 3 | 0 | 0 | 814080 | 0 | 0 | R1 only |
| Osbp | Q3B7Z2 | 4 | 0 | 0 | 805840 | 0 | 0 | R1 only |
| Cbr4 | Q91VT4 | 3 | 0 | 0 | 759080 | 0 | 0 | R1 only |
| Lrrc46 | Q9DAP0 | 5 | 0 | 0 | 736280 | 0 | 0 | R1 only |
| Rel | P15307 | 5 | 0 | 0 | 658640 | 0 | 0 | R1 only |
| Ccdc136 | Q3TVA9 | 8 | 0 | 0 | 566240 | 0 | 0 | R1 only |
| Psma2 | P49722 | 4 | 0 | 0 | 506470 | 0 | 0 | R1 only |
| Zmym4 | A2A791 | 7 | 0 | 0 | 479830 | 0 | 0 | R1 only |
| Dynlt1 | P51807 | 2 | 0 | 0 | 479170 | 0 | 0 | R1 only |
| Snap29 | Q9ERB0 | 2 | 0 | 0 | 478880 | 0 | 0 | R1 only |
| Ssr1 | Q9CY50 | 2 | 0 | 0 | 466850 | 0 | 0 | R1 only |
| Rpn1 | Q91YQ5 | 5 | 0 | 0 | 457730 | 0 | 0 | R1 only |
| Rsph9 | Q9D9V4 | 2 | 0 | 0 | 384920 | 0 | 0 | R1 only |
| Srsf1 | Q6PDM2 | 2 | 0 | 0 | 379540 | 0 | 0 | R1 only |
| Usp28 | Q5I043 | 5 | 0 | 0 | 340430 | 0 | 0 | R1 only |
| Hnrnpa2b1 | O88569 | 2 | 0 | 0 | 334490 | 0 | 0 | R1 only |
| Crybg3 | Q80W49 | 5 | 0 | 0 | 314850 | 0 | 0 | R1 only |
| Pdzd8 | B9EJ80 | 5 | 0 | 0 | 314600 | 0 | 0 | R1 only |
| Ubxn1 | Q922Y1 | 2 | 0 | 0 | 307630 | 0 | 0 | R1 only |
| Rnf220 | Q6PDX6 | 2 | 0 | 0 | 295420 | 0 | 0 | R1 only |
| Klc2 | O88448;O88447 | 4 | 0 | 0 | 294240 | 0 | 0 | R1 only |
| Otud4 | B2RRE7 | 4 | 0 | 0 | 291460 | 0 | 0 | R1 only |
| Wdr61 | Q9ERF3 | 3 | 0 | 0 | 290590 | 0 | 0 | R1 only |
| Mapkap1 | Q8BKH7 | 2 | 0 | 0 | 276910 | 0 | 0 | R1 only |
| Isyna1 | Q9JHU9 | 2 | 0 | 0 | 270340 | 0 | 0 | R1 only |
| Cttnbp2 | B9EJA2 | 6 | 0 | 0 | 252000 | 0 | 0 | R1 only |
| Dld | O08749 | 2 | 0 | 0 | 247930 | 0 | 0 | R1 only |
| Bag6 | Q9Z1R2 | 4 | 0 | 0 | 246860 | 0 | 0 | R1 only |
| Sh3gl2 | Q62420 | 3 | 0 | 0 | 242500 | 0 | 0 | R1 only |
| Hspb1 | P14602 | 2 | 0 | 0 | 241770 | 0 | 0 | R1 only |
| Otub2 | Q9CQX0 | 2 | 0 | 0 | 237760 | 0 | 0 | R1 only |
| Arhgap17 | Q3UIA2 | 3 | 0 | 0 | 237560 | 0 | 0 | R1 only |
| Rpa2 | Q62193 | 3 | 0 | 0 | 235990 | 0 | 0 | R1 only |
| Skiv2l2 | Q9CZU3 | 5 | 0 | 0 | 232690 | 0 | 0 | R1 only |
| Otub1 | Q7TQI3 | 2 | 0 | 0 | 220290 | 0 | 0 | R1 only |
| Rictor | Q6QI06 | 7 | 0 | 0 | 220020 | 0 | 0 | R1 only |
| Atp5f1 | Q9CQQ7 | 2 | 0 | 0 | 212740 | 0 | 0 | R1 only |
| Acot7 | Q91V12 | 2 | 0 | 0 | 190940 | 0 | 0 | R1 only |
| Rdh11 | Q9QYF1 | 2 | 0 | 0 | 183580 | 0 | 0 | R1 only |
| Polr2c | P97760 | 2 | 0 | 0 | 182800 | 0 | 0 | R1 only |
| Rps18 | P62270 | 2 | 1 | 0 | 177970 | 0 | 0 | R1 only |
| Phb2 | O35129 | 4 | 0 | 0 | 150050 | 0 | 0 | R1 only |
| Ccnb1ip1 | D3Z3K2 | 2 | 0 | 0 | 148000 | 0 | 0 | R1 only |
| Cryz | P47199 | 2 | 0 | 0 | 146680 | 0 | 0 | R1 only |
| Aimp2 | Q8R010 | 2 | 0 | 0 | 139050 | 0 | 0 | R1 only |
| Adsl | P54822 | 2 | 0 | 0 | 137730 | 0 | 0 | R1 only |
| Ift122 | Q6NWV3 | 3 | 0 | 0 | 136580 | 0 | 0 | R1 only |
| Fam213a | Q9CYH2 | 2 | 0 | 0 | 128440 | 0 | 0 | R1 only |
| Sugt1 | Q9CX34 | 2 | 0 | 0 | 127610 | 0 | 0 | R1 only |
| Ndufv2 | Q9D6J6 | 3 | 0 | 0 | 127350 | 0 | 0 | R1 only |
| Stx12 | Q9ER00 | 2 | 0 | 0 | 121450 | 0 | 0 | R1 only |
| Wtap | Q9ER69 | 2 | 0 | 0 | 119330 | 0 | 0 | R1 only |
| Fxr1;Fmr1;Fxr2 | Q61584;P35922;Q9WVR4 | 2 | 0 | 0 | 111570 | 0 | 0 | R1 only |
| Pa2g4 | P50580 | 2 | 0 | 0 | 111040 | 0 | 0 | R1 only |
| Cacybp | Q9CXW3 | 2 | 0 | 0 | 110630 | 0 | 0 | R1 only |
| Lrrc59 | Q922Q8 | 2 | 0 | 0 | 110050 | 0 | 0 | R1 only |
| Adad1 | Q5SUE7 | 2 | 0 | 0 | 97389 | 0 | 0 | R1 only |
| Pacs1 | Q8K212 | 2 | 0 | 0 | 96109 | 0 | 0 | R1 only |
| Farsb | Q9WUA2 | 2 | 0 | 0 | 95382 | 0 | 0 | R1 only |
| Ddost | O54734 | 2 | 0 | 0 | 94283 | 0 | 0 | R1 only |
| Ppp1r7 | Q3UM45 | 2 | 0 | 0 | 92202 | 0 | 0 | R1 only |
| Tnrc6b | Q8BKI2 | 3 | 0 | 0 | 91699 | 0 | 0 | R1 only |
| Cul9 | Q80TT8 | 5 | 0 | 0 | 90586 | 0 | 0 | R1 only |
| Nt5c1b | Q91YE9 | 2 | 0 | 0 | 90406 | 0 | 0 | R1 only |
| Nudt16l1 | Q8VHN8 | 2 | 0 | 0 | 86393 | 0 | 0 | R1 only |
| Gart | Q64737 | 2 | 0 | 0 | 84251 | 0 | 0 | R1 only |
| Actl6a;Actl6b | Q9Z2N8;Q99MR0 | 2 | 0 | 0 | 82171 | 0 | 0 | R1 only |
| Prkaca;Prkacb | P05132;P68181 | 2 | 0 | 0 | 82106 | 0 | 0 | R1 only |
| Psmc3 | O88685 | 3 | 0 | 0 | 81001 | 0 | 0 | R1 only |
| Mrps22 | Q9CXW2 | 2 | 0 | 0 | 76739 | 0 | 0 | R1 only |
| Ipo4 | Q8VI75 | 2 | 0 | 0 | 73788 | 0 | 0 | R1 only |
| Psat1 | Q99K85 | 2 | 0 | 0 | 70751 | 0 | 0 | R1 only |
| Get4 | Q9D1H7 | 2 | 0 | 0 | 70049 | 0 | 0 | R1 only |
| Hk1 | P17710;O08528 | 3 | 0 | 0 | 67956 | 0 | 0 | R1 only |
| Cbr1;Cbr3 | P48758;Q8K354 | 2 | 0 | 0 | 67005 | 0 | 0 | R1 only |
| Cab39 | Q06138 | 2 | 0 | 0 | 58078 | 0 | 0 | R1 only |
| Prr5 | Q812A5 | 2 | 0 | 0 | 54652 | 0 | 0 | R1 only |
| Dnajc7 | Q9QYI3 | 2 | 0 | 0 | 48839 | 0 | 0 | R1 only |
| Dhx30 | Q99PU8 | 2 | 0 | 0 | 46771 | 0 | 0 | R1 only |
| Matr3 | Q8K310 | 2 | 0 | 0 | 41923 | 0 | 0 | R1 only |
| Usp9x | P70398 | 2 | 0 | 0 | 24195 | 0 | 0 | R1 only |
| Ctsd | P18242 | 2 | 0 | 0 | 20903 | 0 | 0 | R1 only |
| Myh9;Myh10 | Q8VDD5;Q61879 | 2 | 0 | 0 | 14942 | 0 | 0 | R1 only |

**Supplementary file 1i. crRNAs employed for the generation of the different mouse models.**

| Mouse model | crRNA ID | Sequence |
| --- | --- | --- |
| *Hsf2bp*^-/-^ | sgRNA1 | 5’-TCACAAAACTCTCCATCGTC-3’ |
|  | sgRNA2 | 5’-ATTGGATGGGGATGTCAAGG-3’ |
| *Hsf2bp*^S167L/S167L^ | sgRNA1 | 5’-TCACAAAACTCTCCATCGTC-3’ |
|  | sgRNA2 | 5’-ATTGGATGGGGATGTCAAGG-3’ |
| *Brme1* ^Δ142-472 /Δ142-472^ | sgRNA3 | 5’-AACCTCAGGGACTCTCTCTG-3’ |
|  | sgRNA4 | 5’-GAAGTCTAGTTCCATTGCTG-3’ |
| *Brme1*^-/-^ | sgRNA3 | 5’-AACCTCAGGGACTCTCTCTG-3’ |
| *Spo11*^-/-^ | sgRNA5 | 5’-TATGTCTCTATGCAGATGCA-3’ |
|  | sgRNA6 | 5’- ACACTGACAGCCAGCTCTTT-3’ |
| *Rnf212*^-/-^ | sgRNA7 | 5’- ACCCACGTGAGACTCGCGCG-3’ |
|  | sgRNA8 | 5’- CCTCAAAGGTCCGCGTATTC-3’ |
| *Hei10*^-/-^ | sgRNA9 | 5’- GAAAGGGTACTGTTGCAAGC-3’ |

**Supplementary file 1j. ssODN employed for the different mouse models generation.**

| Mouse model | ssODN sequence |
| --- | --- |
| *Hsf2bp*^S167L/S167L^ | 5’CTTTGGAAAGATGTGACAGTTCTATCTTTTTTATCTTTCAGGACAAAGCATTGAAGTTTTTCAACATAACTGG**A**CAGACGATGGAGAGTTTTGTGAAGT**T**ATTGGATGGGGATGTCAAGGA**A**GTTGATTCTGATGAAAATCAATTTGTCTTTGCACTGGCTGGAATTGTAACAAGTAGGTAACTTTTCAGATACAGCGCT3’ |
| *Brme1* ^Δ142-472 /Δ142-472^ | 5’CTTCAGAGTGCTTGCTTATTGAAGGCCAGGACTGAATCTTCTTTTTCCACAGGAAACAAGGCCAGAGCTGGGAGCCCTCAAAGCAGCCAGCCAGCCACAGGCAATGGAACTAGACTTCCTGCCTGACAGCCAGATACAGGATGCCCTGGATGCCACTAACATGGAGCAGGTAAGAGCTTTCTGTACTCAAATGTACACCC3’ |
| *Spo11^-/-^* | 5’GTTTCCTGCGGTATGTGTTCTCTGCCGTGGTCTGTGTTTGTCACCGTCCAGGAGCAATGCTCATTCTGTGTTGA**G**CTTGCATCTGCATAGAGACATAT**T**CT**T**CACTGACAGCCAGCTCTTTGG**C**AACCAGGCTGCGGTGGACAGCGCCATCGATGACATTTCCTGTATGCTGAAAGTGCCCAGGAGGAGTCTGCACGTGG3’ |

**Supplementary file 1k. Primers and expected product size for genotyping each mutant mouse model.**

| Mouse model | Genotyping Primer | Sequence | Size (bp) |
| --- | --- | --- | --- |
| *Hsf2bp*^-/-^  *Hsf2bp*^S167L/S167L^ | F1 | 5’-TTCTTTGGAAAGATGTGACAGTTC-3’ | WT and KI alelle: 383  KO alelle: 355 |
|  | R1 | 5’-ACCTGGGTTTCCTTTAGATCAGTTA-3’ |  |
| *Brme1 ^Δ142-472/Δ142-472^* | F2 | 5’-GAAAGTTCTTCAGAGTGCTTGCT-3’ | WT alelle: 1228  Δ alelle: 235 |
|  | R2 | 5’-AGCCCTATCTTGTCACCTAAAG-3’ |  |
|  | F3 | 5’-CCCAGCAGATGCCTCTCTTAT-3’ | WT alelle: 150  Δ alelle: No PCR product |
|  | R3 | 5’-CTCAGCAGAGTTCCAATGCAG-3’ |  |
| *Brme1* ^-/-^ | F2 | 5’-GAAAGTTCTTCAGAGTGCTTGCT-3’ | WT alelle: 646  KO alelle: 606 |
|  | R3 | 5’-CTCAGCAGAGTTCCAATGCAG-3’ |  |
| *Spo11*^-/-^ | F4 | 5’- AGAGCCCCCAGTGCTCTTAAC-3’ | WT alelle: 416  KO alelle: 417 |
|  | R4 | 5’- GGCAGACCCCTCTACCTCTGT-3’ |  |
| *Rnf212*^-/-^ | F5 | 5’- TTTCTTTGCCTCCGTACTTTTGG-3’ | WT alelle: 557  KO alelle: 415 |
|  | R5 | 5’- CCCAGGCTTTACTTCAACAACAA -3’ |  |
| *Hei10*^-/-^ | F6 | 5’- CTGCCTGTTCTCACATCTTC-3’ | WT alelle: 100  KO alelle: 96 |
|  | R6 | 5’- AGCTTTCCAGAAAGGGTACTG -3’ |  |

| **Supplementary file 1l. Summary table of all the main and supplementary figures/tables and the associations between them.** |  | | Associated  Suppl. Figures |
| --- | --- | --- | --- |
| Figure 1 | **Pedigree of the consanguineous family with the variant HSF2BP-S167L** | |  |
| Figure 2 | **Mice carrying the HSF2BP S167L variant show a partial reduction of fertility** | | Fig2_S1 |
|  | a | Fertility assessment *Hsf2bp*^S167L/S167L^ females and males |  |
|  | b | Ovaries histology *Hsf2bp*^S167L/S167L^ and *Hsf2bp^-/-^* |  |
|  | c | Follicles quantification *Hsf2bp*^S167L/S167L^ and *Hsf2bp^-/-^* | Fig2_S2a |
|  | d | Testes images *Hsf2bp*^S167L/S167L^ and *Hsf2bp^-/-^* | Fig2_S2b |
|  | e | Testes histology *Hsf2bp*^S167L/S167L^ and *Hsf2bp^-/-^* |  |
|  | f | TUNEL of testis *Hsf2bp*^S167L/S167L^ |  |
|  | g | Epididymis sperm counts *Hsf2bp*^S167L/S167L^ |  |
| Figure 3 | **Meiocytes from *Hsf2bp*^S167L/S167L^ mice show a decrease in the expression of HSF2BP** | |  |
|  | a | HSF2BP staining in *Hsf2bp*^S167L/S167L^ males. Immunofluorescence |  |
|  | b | HSF2BP staining in *Hsf2bp*^S167L/S167L^ females. Immunofluorescence |  |
|  | c | Western blot αHSF2BP in *Hsf2bp*^S167L/S167L^ and *Hsf2bp^-/-^*13 dpp males |  |
| Figure 4 | **DNA repair in *Hsf2bp*** **^S167L/S167L^** **mice** | |  |
|  | a | γH2AX staining in *Hsf2bp*^S167L/S167L^ and *Hsf2bp^-/-^* males. Immunofluorescence |  |
|  | b | RPA1 staining in *Hsf2bp*^S167L/S167L^ and *Hsf2bp^-/-^* males. Immunofluorescence | Fig4_S1a |
|  | c | γH2AX staining in *Hsf2bp*^S167L/S167L^ and *Hsf2bp^-/-^* females. Immunofluorescence |  |
|  | d | RPA1 staining in *Hsf2bp*^S167L/S167L^ and *Hsf2bp^-/-^* females. Immunofluorescence | Fig4_S1b |
| Figure 5 | **The loading of recombinases is compromised in *Hsf2bp*** **^S167L/S167L^** **mice** | |  |
|  | a | DMC1 staining in *Hsf2bp*^S167L/S167L^ and *Hsf2bp^-/-^* males. Immunofluorescence | Fig5_S1a |
|  | b | RAD51 staining in *Hsf2bp*^S167L/S167L^ and *Hsf2bp^-/-^* males. Immunofluorescence | Fig5_S1b |
|  | c | DMC1 staining in *Hsf2bp*^S167L/S167L^ and *Hsf2bp^-/-^* females. Immunofluorescence | Fig5_S1c |
|  | d | RAD51 staining in *Hsf2bp*^S167L/S167L^ and *Hsf2bp^-/-^* females. Immunofluorescence | Fig5_S1d |
| Figure 6 | **Recombination proficiency is decreased in *Hsf2bp*** **^S167L/S167L^** **mice** | |  |
|  | a | SPATA22 staining in *Hsf2bp*^S167L/S167L^ and *Hsf2bp^-/-^* males and females. Immunofluorescence | Fig6_S1a |
|  | b | MLH1 staining in *Hsf2bp*^S167L/S167L^ and *Hsf2bp^-/-^* males. Immunofluorescence | Fig6_S1b |
|  | c | CDK2 staining in *Hsf2bp*^S167L/S167L^ and *Hsf2bp^-/-^* females. Immunofluorescence | Fig6_S1c |
|  | d | Quantification of unsynapsed XY in *Hsf2bp*^S167L/S167L^ and *Hsf2bp^-/-^* males (γH2AX staining) |  |
| Figure 7 | **BRME1, a novel HSF2BP interactor that colocalizes to the recombination nodules** | |  |
|  | a | Coimmunoprecipitation of HSF2BP and BRME1 (HEK293T cells) |  |
|  | b | Testis immunoprecipitation of HSF2BP and BRME1 |  |
|  | c | Coimmunoprecipitation HSF2BP and splitted BRME1 (Nt, central, Ct) |  |
|  | d | HSF2BP and BRME1 colocalization | Supp. File 1d |
|  | e | HSF2BP and BRME1 colocalization STED |  |
|  | f | BRME1 staining in *Hsf2bp*^S167L/S167L^ and *Hsf2bp^-/-^* males. Immunofluorescence | Fig7_S4a |
|  | g | BRME1 staining in *Hsf2bp*^S167L/S167L^ and *Hsf2bp^-/-^* females. Immunofluorescence |  |
|  | h | Western blot αBRME1 in *Hsf2bp*^S167L/S167L^ and *Hsf2bp^-/-^*13 dpp males |  |
| Figure 8 | **BRME1-deficient mice show severe fertility defects** | | Fig8_S1 |
|  | a | Ovaries histology from *Brme1^-/-^*. Follicle counts. |  |
|  | b | Testes image from *Brme1^-/-^* | Fig2_S2b |
|  | c | Testis histology from *Brme1^-/-^* |  |
|  | d | Synapsis analysis in *Brme1^-/-^* males. SYCP3-SYCP1 staining. Immunofluorescence |  |
|  | e | Synapsis analysis in *Brme1^-/-^* females. SYCP3-SYCP1 staining. Immunofluorescence | Fig8_S1e |
|  | f | HSF2BP staining in *Brme1^-/-^* males. Immunofluorescence |  |
|  | g | HSF2BP staining in *Brme1^-/-^* females. Immunofluorescence |  |
|  | h | Western blot αHSF2BP in *Brme1^-/-^*13 dpp males |  |
| Figure 9 | **BRME1 is essential for meiotic recombination** | | **Fig9_S1** |
|  | a | DMC1 staining in *Brme1^-/-^* males. Immunofluorescence | Fig9_S2a |
|  | b | DMC1 staining in *Brme1^-/-^* females. Immunofluorescence | Fig9_S2b |
|  | c | RAD51 staining in *Brme1^-/-^* males. Immunofluorescence | Fig9_S2c |
|  | d | RAD51 staining in *Brme1^-/-^* females. Immunofluorescence | Fig9_S2d |
|  | e | SPATA22 staining in *Brme1^-/-^* males. Immunofluorescence (just quantification plots) | Fig9_S2e |
|  | f | SPATA22 staining in *Brme1^-/-^* females. Immunofluorescence (just quantification plots) | Fig9_S2f |
|  | g | MLH1 staining in *Brme1^-/-^* males. Immunofluorescence |  |
|  | h | CDK2 staining in *Brme1^-/-^* females. Immunofluorescence |  |
| Figure 10 | **BRME1 forms a complex with BRCA2 and HSF2BP and stabilizes HSF2BP** | | **Fig10_S1** |
|  | a | Negative coimmunoprecipitations between BRME1 and splitted BRCA2 (Nt, medium, Ct) |  |
|  | b | Triple coimmunoprecipitation between BRME1, BRCA2-C and HSF2BP (HEK293T cells) |  |
|  | c | Cotransfection of BRME1 and HSF2BP in U2OS cells. Immunofluorescence. |  |
|  | d | Western blot analysis of HSF2BP stabilization by BRME1 (HEK293T cells) |  |

|  |  | Associated  Main figures |
| --- | --- | --- |
| Figure 1_S1 | Segregation of the Ser167Leu Variant in HSF2BP in the consanguineous family |  |
| Figure 1_S2 | Strong conservation of the Ser167 residue in HSF2BP protein in 99 mammals |  |
| Figure 1_S3 | Strong conservation of the Ser167 residue in HSF2BP in 48 birds and reptiles and 64 fish species |  |
| Figure 2_S1 | Generation and genetic characterization of Hsf2bp Ser167Leu and Hsf2bp-deficient mice | 2 |
| Figure 2_S2 | Fertility defects in *Hsf2bp*^S167L/S167L^ mice | 2 |
| Figure 3_S1 | *Hsf2bp*^S167L/S167L^ mice do not show synapsis defects | 3a |
| Figure 4_S1 | RPA1 localization in *Hsf2bp* mutants | 4b, 4d |
| Figure 5_S1 | Defective loading of recombinases in *Hsf2bp*^S167L/S167L^ mice | 5 |
| Figure 6_S1 | Meiotic recombination is affected in *Hsf2bp*^S167L/S167L^ mice | 6 |
| Figure 6_S2 | Comparative interaction of HSF2BP-S167L and HSF2BP-WT with BRCA2 |  |
| Figure 7_S1 | *Brme1* Δ142-472 mutants do not show meiotic defects | 7c |
| Figure 7_S2 | C19ORF57/BRME1 localizes at meiotic recombination nodules | 7d-e |
| Figure 7_S3 | Colocalization analysis of HSF2BP and BRME1 with RPA and DMC1 | Supp.Files 1d-1e |
| Figure 7_S4 | BRME1 localization depends on HSF2BP and any of them has DNA binding abilities | 7f-g |
| Figure 7_S5 | Generation and genetic characterization of *Rnf212^-/-^* and *Hei10^-/-^* mice |  |
| Figure 7_S6 | BRME1 loading depends on DSBs generation but not on synapsis |  |
| Figure 8_S1 | Generation and genetic characterization of *Brme1* knock-out mice | 8 |
| Figure 9_S1 | DSBs are formed but not properly repaired in *Brme1*-deficient mice and mimic the phenotype of *Hsf2bp*-deficient mice | 9 |
| Figure 9_S2 | Altered dynamic of recombinational proteins in the absence of BRME1 | 9a-f |
| Figure 10_S1 | Co-immunoprecipitations of BRME1, HSF2BP, RPA, RAD51 and PALB2 expressed from transfected HEK293 cells and from TNT assays | 10 |

|  |  | Associated  Figures |
| --- | --- | --- |
| Supp.File 1a | Whole Exome Sequencing and mapping data for the POI patients and the unaffected sister |  |
| Supp.File 1b | Filtering of the variants identified by Whole Exome Sequencing |  |
| Supp.File 1c | Pathogenicity predictions for the S167L variant in *HSF2BP* |  |
| Supp.File 1d | Quantification of HSF2BP colocalization with RPA, DMC1 and BRME1 | 7d, Fig7_S3 |
| Supp.File 1e | Quantification of BRME1 colocalization with RPA and DMC1 | Fig7_S3 |
| Supp.File 1f | Summary table comparing the different molecular alterations on each genotype for the different meiotic players analyzed | 4,5,6,9 |
| Supp.File 1g | Meiotic recombination players identified as putative BRME1 interactors by IP-MS of whole testis extracts with two antibodies (R1 and R2) against BRME1 |  |
| Supp.File 1h | Extended data of BRME1 putative interactors identified by IP-MS of whole testis extracts with two different antibodies (R1 and R2) against BRME1 |  |
| Supp.File 1i | crRNAs employed for the generation of the different mouse models | Fig2_S1,Fig7_S5, Fig7_S6,Fig8_S1 |
| Supp.File 1j | ssODN employed for the different mouse models generation | Fig2_S1, Fig8_S1 |
| Supp.File 1k | Primers and expected product size for genotyping each mutant mouse model | Fig2_S1,Fig7_S5, Fig7_S6,Fig8_S1 |
| Supp.File 1l | Summary table of all the main and supplementary figures/tables and the associations between them |  |
